# High-Voltage Electrostatic Field Hydrogel Microsphere 3D Culture System Improves Viability and Liver-like Properties of HepG2 Cells

**DOI:** 10.3390/ijms25021081

**Published:** 2024-01-16

**Authors:** Yi Liu, Yang Ge, Yanfan Wu, Yongtong Feng, Han Liu, Wei Cao, Jinsong Xie, Jingzhong Zhang

**Affiliations:** 1 School of Biomedical Engineering (Suzhou), Division of Life Sciences and Medicine, University of Science and Technology of China, Hefei 230026, China; liuyi@sibet.ac.cn (Y.L.); wuyf@sibet.ac.cn (Y.W.); 2 The CAS Key Laboratory of Bio-Medical Diagnostics, Suzhou Institute of Biomedical Engineering and Technology, Chinese Academy of Sciences, Suzhou 215163, China; gey@sibet.ac.cn (Y.G.); fengyt@sibet.ac.cn (Y.F.); liuhan@sibet.ac.cn (H.L.); caow@sibet.ac.cn (W.C.); xiejs@sibet.ac.cn (J.X.); 3 School of Medical Imaging, Xuzhou Medical University, Xuzhou 221004, China

**Keywords:** hepatocyte, 3D cultures, hydrogel, microspheres, high-voltage electrostatic field, RNA-seq

## Abstract

Three-dimensional (3D) hepatocyte models have become a research hotspot for evaluating drug metabolism and hepatotoxicity. Compared to two-dimensional (2D) cultures, 3D cultures are better at mimicking the morphology and microenvironment of hepatocytes in vivo. However, commonly used 3D culture techniques are not suitable for high-throughput drug screening (HTS) due to their high cost, complex handling, and inability to simulate cell–extracellular matrix (ECM) interactions. This article describes a method for rapid and reproducible 3D cell cultures with ECM–cell interactions based on 3D culture instrumentation to provide more efficient HTS. We developed a microsphere preparation based on a high-voltage electrostatic (HVE) field and used sodium alginate- and collagen-based hydrogels as scaffolds for 3D cultures of HepG2 cells. The microsphere-generating device enables the rapid and reproducible preparation of bioactive hydrogel microspheres. This 3D culture system exhibited better cell viability, heterogeneity, and drug-metabolizing activity than 2D and other 3D culture models, and the long-term culture characteristics of this system make it suitable for predicting long-term liver toxicity. This system improves the overall applicability of HepG2 spheroids in safety assessment studies, and this simple and controllable high-throughput-compatible method shows potential for use in drug toxicity screening assays and mechanistic studies.

## 1. Introduction

The liver plays a crucial role in drug metabolism and consists of two main cell types: parenchymal and nonparenchymal cells. Parenchymal cells, such as hepatocytes and bile duct cells, constitute approximately 70% and 3–5% of the total hepatocyte population, respectively [1]. Nonparenchymal cells (NPCs) include Kupffer cells, hepatic endothelial cells, and hepatic stellate cells, accounting for approximately 25% of the total hepatocyte population [2]. As the body’s primary detoxification organ, the liver metabolizes most exogenous drugs into nontoxic or less toxic substances. However, certain drugs or their metabolites can cause liver damage, leading to the discontinuation of drug development or withdrawal of a marketed drug [3].

During drug development, in vitro hepatocyte models are commonly used to test the cytotoxicity of prodrugs [4]. The origin of the cells selected for these models is crucial to the relevance and predictive value of these models. To date, primary human hepatocytes (PHHs) have been considered to be the gold standard for hepatocyte models, as they directly reflect the specific metabolism and function of the human liver. Moreover, human pluripotent stem-cell-derived hepatocyte-like cells (hPSC-HLCs) have also had a tremendous number of applications in recent decades. However, the scarcity of PHHs and logistical challenges associated with obtaining them have led researchers to explore alternative cell sources. hPSC-HLCs may be a valid alternative for PHH-based hepatocyte models; nevertheless, protocols to generate hPSC-HLCs produce cells either with hybrid features of hepatic, intestinal, fibroblastic, and pluripotent cells or that are similar to fetal hepatocytes [5]. Hepatocytes derived from hepatocellular carcinoma or that have undergone genetic manipulation (especially HepG2 cells) are widely utilized due to their unlimited lifespan and widespread availability [6,7,8].

In conventional 2D cultures, hepatocytes often fail to replicate the in vivo microenvironment [9] and exhibit altered metabolic functions, such as low expression of cytochrome P450 (CYP450) enzymes [10] and xenobiotic receptors that regulate the expression of drug-metabolizing enzymes [11,12]. The low expression of these metabolic enzymes in hepatocyte lines is an important factor contributing to poor drug toxicity predictions. Furthermore, rapid hepatocyte dedifferentiation in 2D cultures results in the loss of their liver phenotype and function, rendering them of limited utility as alternatives to animal models [13]. As a result, the drug response levels and analytical data obtained from this model are biased and a substantial number of early drug candidates fail in later clinical trials [14].

In contrast to 2D models, 3D culture systems consisting of hepatocytes establish intercellular and cell–ECM interactions that closely resemble or even replicate those found in native tissue [15]. Three-dimensional hepatocyte culture models better reflect in vivo liver physiology and are more similar in terms of cellular heterogeneity, morphology, material transport, and biotransformation activity to cells in vivo [16,17]. As a result, 3D hepatocyte models have emerged as a promising tool for the assessment of drug metabolism and toxic effects. Moreover, incorporating 3D-cultured cells into HTS enables rapid assessments of drug candidates in vitro and in vivo [18], leading to cost reductions in drug development and a reduced need for animal testing [19]. In the past decade, numerous studies have focused on developing and optimizing various 3D culture strategies to preserve liver properties in vitro [20]. Currently, scaffold-based systems, bioreactors, and on-chip tumor approaches have limited applicability in HTS [21], while scaffold-free systems, including ultra-low adhesion plates (ULAPs) [22], hanging droplet plates (HDs) [23], and hydrogel and microcarrier 3D cell culture systems, are suitable for HTS applications. However, simple 3D culture techniques, such as ULAPs and HDs, are unable to simulate cell–ECM interactions, which are important in supporting cell proliferation and differentiation [24]. Superior 3D culture techniques, such as hydrogels and scaffolds, need complex handling [25]. Therefore, a simple method for 3D hepatocyte modeling with an ECM microenvironment that can be used for HTS screening is urgently needed.

**Figure 1:**
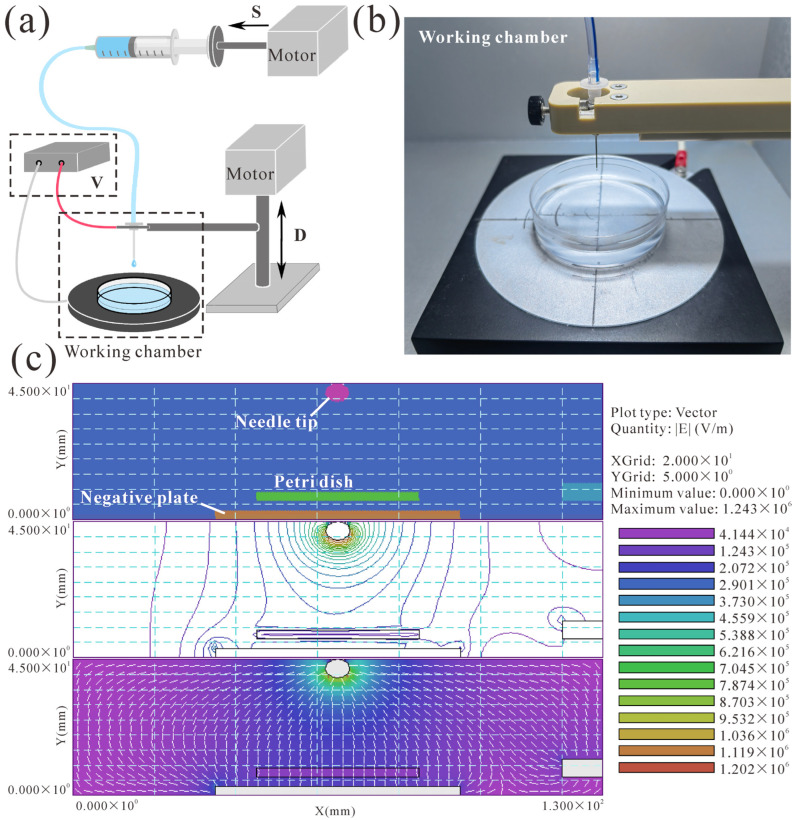
(**a**) Schematic diagram of the microsphere generation device, the liquid surface (D), the injection speed (S), and the voltage (V), which were changed using high-voltage power supply (HVPS) and motor; (**b**) the internal structure of the working chamber, including the injection needle connected to the positive terminal of the HVPS, the negative plate, and the petri dish containing the calcium chloride solution; and (**c**) simulation showing the electric field between the needle tip and the negative plate generated by the high-voltage power supply.

In this study, we developed a composite hydrogel, RGD (Arg-Gly-Asp) peptide-grafted oxidized sodium alginate hydrogel (ROSAH), to simulate the ECM microenvironment in vivo using HepG2 cells. RGD adhesion peptides have minimal recognition sequences required for cell adhesion [26]. It has been shown that the RGD domains in the matrix are necessary for organoid growth [27]. Compared to scaffolds without RGD, hydrogels doped with RGD support hepatocyte viability and function [5,28]. Oxidized alginate (OA) is commonly used in hydrogel and microsphere materials due to its desirable degradation rate and properties [29]. Furthermore, grafting RGD peptides onto OAs can enhance cell adhesion and adherence and influence the metabolic function of hepatocytes [5]. The high-voltage electrostatic (HVE) droplet method is widely used for the preparation of microspheres with uniform particle sizes, smooth surfaces, and simple operational procedures [30,31]. We used HepG2 cells embedded in ROSAH + collagen to prepare hydrogel microspheres (RCHMs) using a 3D microsphere generation device based on an HVE field. (Figure 1). We investigated the effects of varying the voltage, distance between the needle tip and the liquid surface, and injection speeds on the size of the microspheres. By adjusting the three parameters above, we can rapidly prepare RCHMs of any diameter between 200 and 1500 μm. Additionally, we screened hydrogels for optimal pore size, oxidation levels, and engrafted RGD concentrations to enhance HepG2 cell viability. HepG2 cells cultured with RCHMs were compared to 2D and other 3D cultures. We quantified their cell viability, metabolic and secretory capabilities, drug sensitivity, and expression levels of genes encoding liver-specific markers to determine which system exhibited the most liver-like properties. By designing a model that realistically simulates biological liver processes, we aimed to improve liver characteristics such as the viability, heterogeneity, and drug metabolism activity of in vitro hepatocyte spheroids in order to increase the reliability and accuracy of predictions of experimental results. The RCHM system improves the overall applicability of HepG2 spheroids in safety assessment studies, and this simple and controllable high-throughput-compatible method has potential for use in drug toxicity screening assays and mechanistic studies.

## 2. Results

### 2.1. Regulation of Microsphere Size

**Figure 2:**
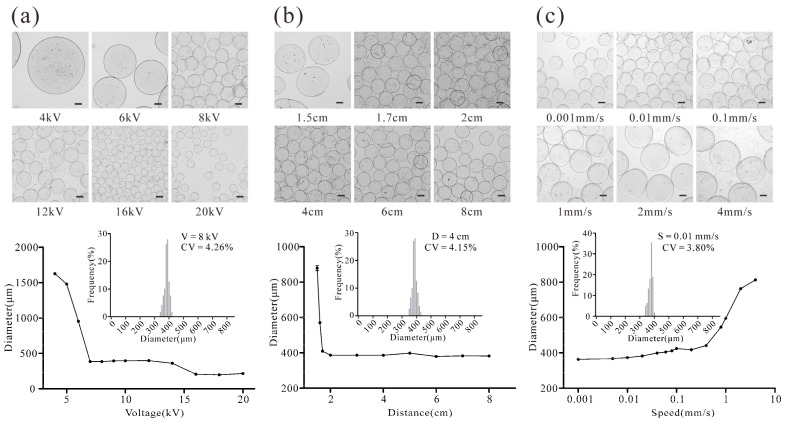
Size of microspheres when different parameters were used. (**a**) The electric field force was changed and affected the size of microspheres when varying the voltage. (**b**) The working distance was varied to change the electrostatic field and affected the size of microspheres. (**c**) Effect of injection speed on the size of microspheres. The microspheres were analyzed via light microscopy (scale bar: 200 µm).

To establish a hepatocyte model that better reflects liver tissue to enable more effective drug screening, we first aimed to simplify and control the preparation steps of hydrogel microspheres supporting 3D cultures of HepG2 cells. The microsphere generation device produces microspheres of varying particle sizes when parameters such as the distance between the needle tip and the liquid surface (D), the injection speed (S), and the voltage (V) were changed. To examine the influence of these parameters on the microsphere size, a particle size analysis was conducted on microspheres generated under different conditions. As shown in Figure 2a, the particle size of the microspheres exhibited an overall decrease with increasing voltage. The diameter size decreased sharply within the range of 4–6 kV, reaching approximately 400 μm at 7–14 kV, and further decreased to less than 200 μm at 16–20 kV. When the voltage was set to 8 kV, the microsphere diameter was measured as 383.98 ± 1.50 μm, whereas at 18 kV, the microsphere diameter was found to be 198.26 ± 0.83 μm. The primarily effect on microsphere size was observed when the distance between the needle tip and the liquid surface was in the range of 1.5~2 cm, as shown in Figure 2b. Moreover, the particle size of the microspheres was found to be directly proportional to the injection speed. Notably, the microsphere diameter showed minimal variation within an injection rate range of 0.001–0.4 mm/s, and the particle size increased significantly when the injection rate was between 0.8 and 4 mm/s (Figure 2c). Based on these findings, the following instrument parameters were considered optimal for enabling the rapid and stable preparation of microspheres: V = 8 kV, D = 4 cm, and S = 0.01 mm/s (coefficient of variation (CV) < 5%).

### 2.2. Generating Material with Differential Pore Sizes

**Figure 3:**
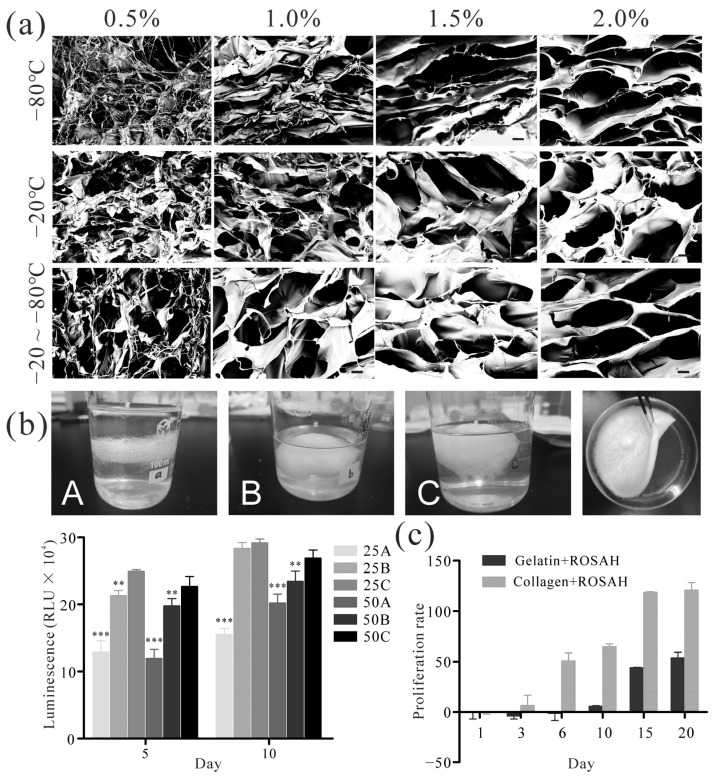
Preparation of hydrogel materials. (**a**) Hydrogel materials were prepared with different concentrations of sodium alginate and cooling methods, and the three-dimensional structures were observed via scanning electron microscopy (scale bar: 40 µm). (**b**) Bioactive hydrogels were prepared by adding high (A: 1000 µL), medium (B: 500 µL), and low (C: 250 µL) doses of sodium periodate (0.25 M) to 1% sodium alginate solution and grafting 25 μg/mL and 50 μg/mL of Arg-Gly-Asp (RGD) peptide, respectively. Effects of degree of hydrogel oxidation and engrafted RGD concentration on the viability of HepG2 cells were compared. (**c**) Effect of adding gelatin or collagen to RGD peptide-grafted oxidized sodium alginate hydrogel (ROSAH) on hepatocyte proliferation (*n* = 5; ** *p* < 0.01 and *** *p* < 0.001 relative to 25C group).

The hydrogel material used to generate microspheres is crucial for hepatocyte modelling, in which the pores of the material influence cell attachment, growth, and the diffusion of oxygen, nutrients, and metabolites. Different concentrations of oxidized sodium alginate form a material with different-sized pores after lyophilization. Furthermore, the cooling conditions can influence the porosity and regularity of the material. Among the three cooling conditions analyzed, the lyophilized material obtained with 0.5% sodium alginate exhibited smaller and irregular pore sizes, while the material derived from 2% sodium alginate displayed the largest and more regular pore sizes (Figure 3a). Specifically, the cooling method involving initial cooling at −20 °C for 2 h followed by freezing at −80 °C yielded material with more uniform and regular pores compared to those of the other two methods. Interestingly, there was no significant difference in the pore size of the 1.0% (168.48 ± 6.93 μm), 1.5% (172.59 ± 7.78 μm), and 2.0% (167.96 ± 6.20 μm) lyophilized materials obtained with the aforementioned cooling method. Based on these observations, the final approach for pore production was determined to be 1.0% oxidized sodium alginate cooled at −20 °C for 2 h followed by freezing at −80 °C.

### 2.3. Bioactivity of Sodium Alginate

To better simulate the in vivo microenvironment and cell–ECM interactions in vitro, the impact of different degrees of oxidation and grafted RGD concentrations on the viability of 3D-cultured HepG2 cells were investigated as measured via a quantification of ATP (CellTiter-Glo). Three different volumes of sodium periodate were used to prepare high, medium, and low oxidation levels of sodium alginate: (A) 1000 µL, (B) 500 µL, and (C) 250 µL. Additionally, the effect of 25 µg/mL and 50 µg/mL RGD engraftment was analyzed. Specifically, six different materials were analyzed. As shown in Figure 3b, a higher degree of oxidation led to a lower level of cell viability. Furthermore, the effect of the two RGD concentrations on the cell viability was found to be nonsignificant. Among them, the highest cell viability was observed in the sodium alginate with low oxidization levels (C: 250 µL) when the RGD grafting concentration was 25 µg/mL, with luminescence values of (24.90 ± 0.25) × 10^4^ RLU and (29.18 ± 0.59) × 10^4^ RLU on days 5 and 10, respectively. Thus, the optimal bioactive hydrogels were selected for engraftment at an RGD concentration of 25 µg/mL and sodium alginate oxidized with 250 µL of sodium periodate. These findings highlight the importance of the degree of oxidation for cell viability. The selected parameters demonstrating the most favorable outcomes for cell growth.

### 2.4. Collagen Promotes Cell Proliferation in Hydrogels

The activity of cells within the material is also influenced by the formulation of bioactive hydrogels. Therefore, we compared the proliferation of cells in two hydrogels, one containing collagen and one containing gelatin (Figure 3c). The results revealed no significant difference in the proliferation rate of HepG2 cells in the bioactive hydrogels containing collagen or gelatin until day 3. However, starting on day 6, the rate of cell proliferation in the collagen + ROSAH group was significantly higher than that in the gelatin + ROSAH group. On days 15 and 20, the percentages of proliferating cells in the collagen + ROSAH group reached 118.93 ± 0.57% (*p* < 0.001) and 121.02 ± 7.49% (*p* < 0.01), respectively, which were significantly higher than the values observed in the gelatin + ROSAH group (44.02 ± 0.53% and 53.88 ± 5.53%). These results indicate that bioactive hydrogels containing collagen are more favorable for the growth of HepG2 cells than bioactive hydrogels containing gelatin.

### 2.5. Differences in Hepatocyte Proliferation Rates in Different Culture Models

**Figure 4:**
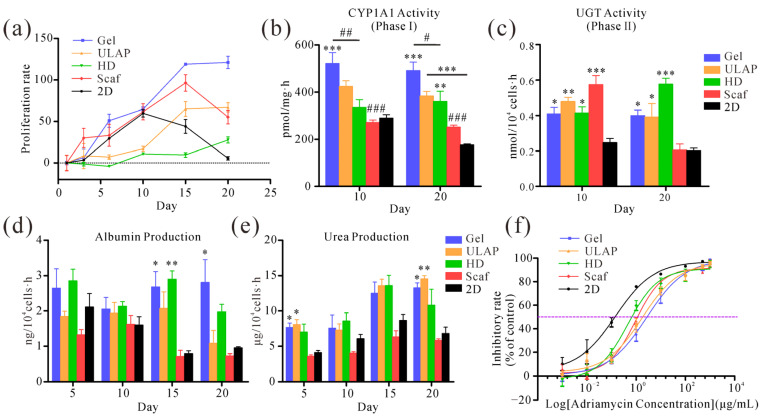
Enhanced viability, metabolic and biotransformation competence, and lower drug sensitivity of HepG2 cells in the RCHM (Gel) culture system. (**a**) Proliferation of HepG2 cells in 3D versus 2D culture models. (**b**) EROD activity (phase I), (**c**) UGT activity (phase II), and (**d**) albumin production and (**e**) urea secretion in various culture models. (**f**) Drug sensitivity of different hepatocyte models, the purple dotted line represents 50% inhibitory rate (*n* = 4; * *p* < 0.05, ** *p* < 0.01 and *** *p* < 0.001 relative to 2D; # *p* < 0.05, ## *p* < 0.01 and ### *p* < 0.001 relative to Gel).

The proliferation of HepG2 cells in the four 3D culture models and the 2D culture model is depicted in Figure 4a. The HepG2 cells cultured in hydrogels exhibited a rapid proliferation over time, reaching the highest growth rate (121.02 ± 7.49%) on day 20. In contrast, the HepG2 cells in 2D and PS fibrous scaffolds (Scaf) cultures exhibited a significant decrease in cell proliferation at days 10 and 15, respectively, indicating that these cells are unsuitable for long-term cultures. Additionally, the HepG2 cells cultured on ULAPs and HDs displayed a lower proliferation rate over time. The HD group exhibited the lowest rate of cell proliferation (27.82 ± 3.63%) on day 20. On the other hand, HepG2 cells cultured on ULAPs demonstrated an increasing proliferation rate from day 10 (17.12 ± 3.17%) to day 15 (65.07 ± 8.83%), remaining stable at 67.04 ± 5.57% by day 20. In conclusion, 2D and Scaf were found to be unsuitable for the long-term culture of HepG2 cells. The HepG2 cells cultured using the HD model exhibited a low proliferation rate. The HepG2 cells cultured on ULAPs and hydrogels were deemed suitable for long-term cultures, although the proliferation rate of the HepG2 cells cultured on ULAPs was lower than that of those grown on hydrogels. This difference may be attributed to the lack of extracellular matrix support and adhesion sites in the ULAPs.

### 2.6. Biotransformation Activity

As depicted in Figure 4b, after 10 days of culture, the hepatocyte spheroids in the hydrogel group exhibited the highest ethoxyresorufin-O-deethylase (EROD) activity, which was significantly greater than that in the HD, Scaf, and 2D culture groups (*p* < 0.01). The EROD activity in the HD and Scaf groups was similar to that in the 2D-cultured hepatocyte group on day 10. After 20 days in culture, the hepatocyte spheroids in all the 3D culture groups except for the Scaf group demonstrated significantly higher levels of EROD activity than the 2D culture group (*p* < 0.01). Furthermore, the ability of uridine 5’-diphosphate glucuronosyltransferase (UGT) to convert 4-methylumbelliferone (4-MU) represented its phase II metabolic capacity (Figure 4c). The hepatocyte models in all 3D culture systems exhibited higher levels of UGT activity than the 2D culture on day 10 (*p* < 0.05). By day 20, the UGT activity level of the hepatocyte spheroids in the 3D culture systems remained significantly higher than that in the 2D culture group, except for that in the Scaf culture group. The lack of a significant difference between the Scaf and 2D culture groups may be attributed to the prolonged culture period leading to apoptosis or necrosis. In summary, compared with those in the 2D culture system, the hepatocyte spheroids cultivated in the hydrogel, ULAP, and HD culture systems exhibited superior phase I and phase II metabolism. Moreover, these 3D culture systems enabled the maintenance or enhancement of enzymatic activity over time.

### 2.7. Albumin Secretion and Urea Synthesis

After 5 and 10 days of culture, the cells in all culture model systems exhibited comparable albumin secretion capacities (Figure 4d). However, on day 15, hepatocyte spheroids in the hydrogel and HD groups demonstrated higher levels of albumin synthesis than those in the Scaf or 2D culture groups (*p* < 0.05). Furthermore, on day 20, the hydrogel group exhibited a significantly superior albumin synthesis capacity compared to that of the ULAP, Scaf, and 2D culture groups (*p* < 0.05). Additionally, hepatocyte spheroids grown in the hydrogel, ULAP, and HD culture systems displayed enhanced metabolic characteristics in terms of urea production compared to those in the 2D culture group (Figure 4e). In particular, the hydrogel and ULAP groups exhibited significantly greater urea production than the 2D culture group on days 5 and 20 (*p* < 0.05). In contrast, the Scaf group exhibited similar levels of urea synthesis to that of the 2D-cultured hepatocyte model. In conclusion, prolonged Scaf and 2D cultures resulted in decreased albumin and urea production in HepG2 cells. On the other hand, HepG2 cells cultured on ULAPs showed increased urea synthesis. Notably, the hydrogel culture promoted both urea secretion and albumin synthesis in HepG2 cells, indicating the effective metabolic function of the cells.

### 2.8. Drug Sensitivity Analysis

The 2D and 3D hepatocyte culture models were exposed to doxorubicin at concentrations ranging from 0 to 1000 μg/mL for 48 h. As depicted in Figure 4f, the cells in the 2D culture exhibited a higher level of drug sensitivity (half-maximal inhibitory concentration (IC50) = 0.16 ± 0.07 μg/mL) compared to that of the 3D hepatocyte culture model. Among the 3D culture systems, hepatocyte spheroids cultured in the HD and Scaf systems demonstrated relatively higher levels of sensitivity to doxorubicin, with IC50 values of 0.46 ± 0.12 μg/mL and 0.92 ± 0.16 μg/mL, respectively. In contrast, hepatocyte spheroids cultured on ULAPs and the hydrogel exhibited lower drug sensitivity levels with IC50 values of 1.83 ± 0.62 μg/mL and 2.40 ± 0.81 μg/mL, respectively.

### 2.9. Cell Viability in Different 3D Models

**Figure 5:**
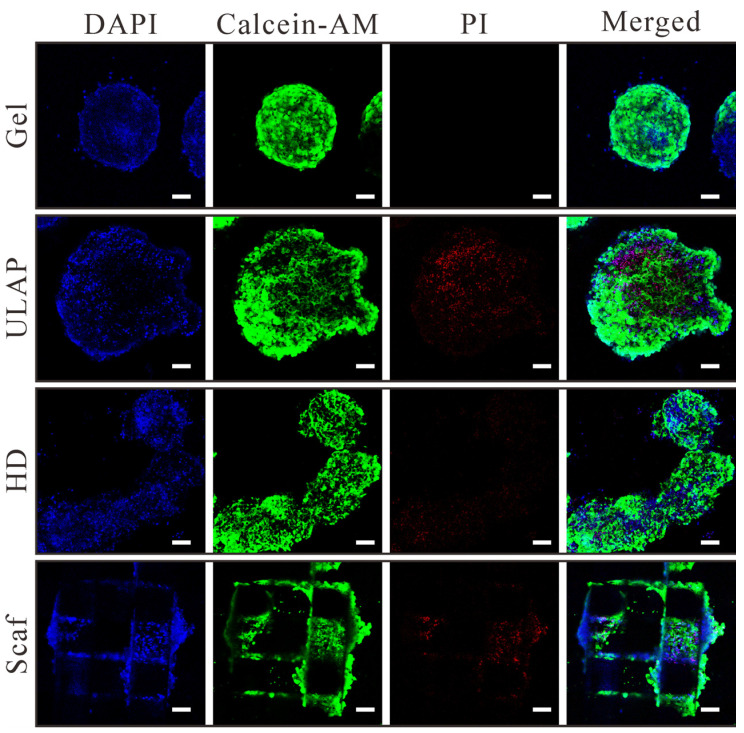
Live and dead HepG2 cells were stained using Calcein-AM /PI in different 3D culture models, the viable cells were stained with Calcein-AM (green), while the dead cells were stained with PI (red), and the nuclei were stained with DAPI (blue) (scale bar: 75 µm).

Calcein-AM/PI (propidium iodide) staining was used to measure the cell viability and was observed via laser confocal microscopy. The HepG2 cells in the hydrogel formed smaller-diameter cell clusters with a compact structure and did not induce necrosis. In contrast, the ULAP and HD models produced mainly single hepatocyte spheres (Figure 5). The diameter of the hepatocyte spheres that formed in the ULAP model was large, leading to hypoxia-induced necrosis in the center of the spheroid. On the other hand, the cell clusters formed in the HD model displayed irregular shapes and partial necrosis. In the case of the Polystyrene (PS) fibrous scaffold, HepG2 cells exhibited a 2.5D growth pattern, with some cells aligning along the scaffold walls and others forming clusters at the intersections of the PS fibers. After prolonged cultures, the cells on the scaffold exhibited apoptosis and necrosis.

### 2.10. RNA-Seq Analysis

**Figure 6:**
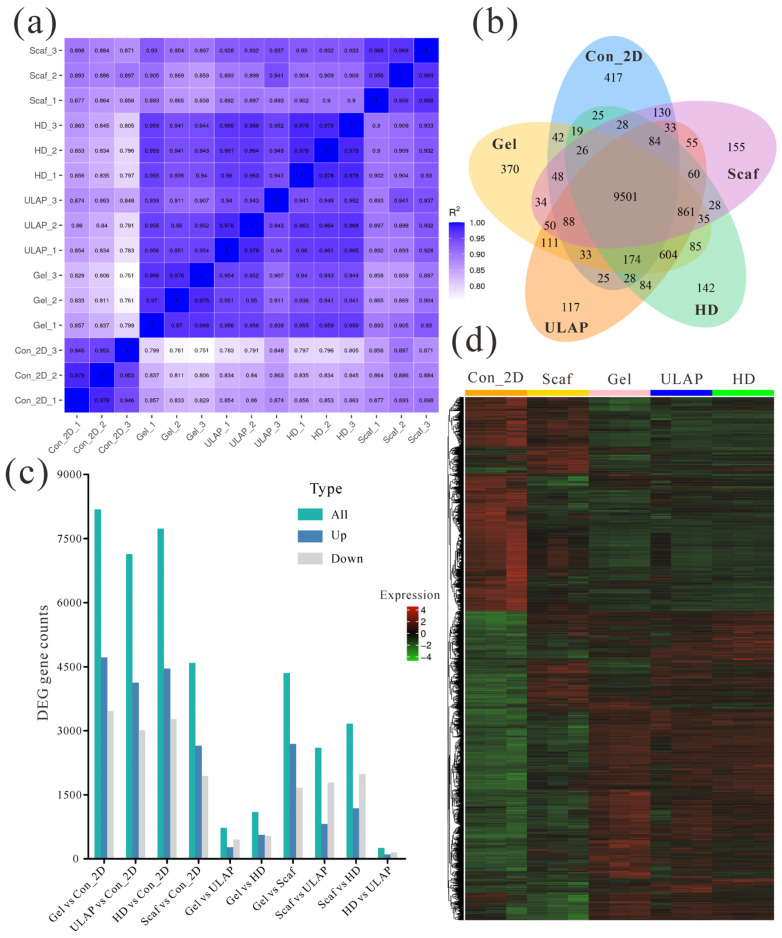
Analysis of gene expression profiles of various HepG2 cell models. (**a**) Correlation heatmap of samples; (**b**) co-expression Venn diagram; (**c**) histogram showing the number of different combinations of DEGs; and (**d**) heatmap showing differentially expressed gene clusters (3 biological replicates per group).

We visualized the correlations of each sample in a heatmap (Figure 6a) based on the Pearson correlation coefficient (R2). The heatmap revealed that the correlation coefficients for the Gel (RCHM), ULAP, and HD groups (R2 ≥ 0.907) were significantly higher than those for the Con_2D (2D Control) and Scaf groups. The correlation coefficients suggest that the expression patterns of genes in the HepG2 cells in these 3D culture models were largely similar. Notably, the hepatocyte genes in the Gel and HD groups exhibited the highest intragroup correlation coefficients (R2 ≥ 0.968), indicating excellent sample reproducibility. Furthermore, a coexpression Venn diagram (Figure 6b) revealed the number of uniquely expressed genes in each group/sample. Interestingly, 604 genes were coexpressed in the Gel, ULAP, and HD groups, which was significantly higher than the number of genes coexpressed between each of these groups and the Con_2D or Scaf groups. We normalized the raw read count using DESeq and performed hypothesis testing to calculate the probability value (*p* value) while correcting for multiple hypothesis testing. Figure 6c presents the statistics regarding the number of differentially expressed genes (DEGs, both upregulated and downregulated) for each combination among the five experimental groups that were compared. Notably, the Gel vs. ULAP, Gel vs. HD, and HD vs. ULAP comparisons revealed the fewest DEGs, with 726 (upregulated: 272, downregulated: 454), 1098 (upregulated: 563, downregulated: 535), and 252 (upregulated: 102, downregulated: 150), respectively. These results further support the notion that the gene expression pattern of HepG2 cells cultured in Gel is more similar to that of these cells cultures on ULAPs and HDs. Finally, a clustering analysis confirmed this finding. The heatmap (Figure 6d) demonstrates that the Gel, ULAP, and HD groups exhibited a tightly clustered pattern, distinguishing them from the 2D and Scaf culture groups.

**Figure 7:**
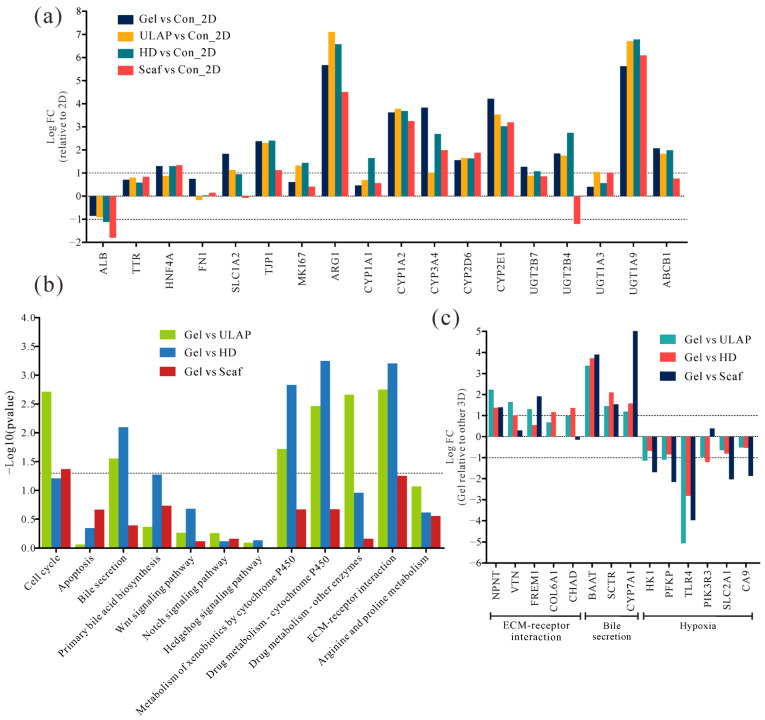
Differential expression of marker genes among different hepatocyte models. (**a**) Fold change in gene expression between 3D and 2D cell models. (**b**) KEGG pathway enrichment analysis showing the annotation of important liver pathways and (**c**) fold change of the expression of DEGs in the hydrogel compared to the other 3D models. Three biological replicates per group; |log2 (fold change)| ≥ 1 *p* ≤ 0.05 were the thresholds indicating a significant difference between cellular models.

In addition, we analyzed and compared the expression of a range of liver-specific and metabolism-related genes in HepG2 cells under different culture conditions. Compared to the genes expressed in cells in the 2D culture system, the expression of functional hepatocyte markers was preserved during prolonged cultures in the 3D system, and the levels of most hepatocyte markers were significantly increased in the 3D culture system (Figure 7a). Specifically, hepatocyte nuclear factor 4α (*HNF4α*), hepatocyte epithelial marker (*TJP1*), urea synthesis (*ARG1*), phase I metabolic enzymes (CYP1A2, CYP3A4, CYP2D6, and CYP2E1), and phase II xenobiotic metabolizing enzymes (UGT1A9) exhibited notable increased expressions in the 3D culture system. On the other hand, the protein expression of the phase II metabolizing enzyme UGT2B4 was lower in the Scaf culture group than in the other 3D culture systems, and it was 1.20-fold lower than that in the 2D culture system. This difference may explain the decrease in UGT enzyme activity observed in the Scaf group after 20 days of culture (Figure 4c). The lower level of ALB gene expression in the 3D culture system than in the 2D culture system was expected, as HepG2 cells cultured in the 2D culture system for 5 days exhibited higher levels of ALB secretion (Figure 4d). Furthermore, the expression of *ABCB1* genes, which have been associated with tumor multidrug resistance, was increased in the 3D culture systems compared to that of the 2D culture system. This finding aligns with the observation that the 3D hepatocyte culture models displayed lower levels of sensitivity to doxorubicin in the drug sensitivity assay.

To investigate the impact of the Gel culture system on the biological processes of HepG2 cells compared to that of other 3D culture systems, we mapped all the DEGs to KEGG pathways and identified 12 important hepatic pathways [32] (Figure 7b). The results revealed a significant enrichment of DEGs in the pathways related to cell cycle, bile secretion, drug and xenobiotic metabolism, and extracellular matrix (ECM)–receptor interaction in the Gel culture system (*p* < 0.05). A differential expression analysis of the scaffold-based 3D culture systems (the Gel and Scaf systems) revealed a higher expression of ECM receptor pathway-related genes than that of scaffold-free culture systems (the ULAP and HD systems) (Figure 7c). Moreover, HepG2 cells in the Gel culture system displayed the lowest drug sensitivity, which is attributed to the ability of the ECM to mediate cellular drug resistance [33,34]. Additionally, the expression of bile secretion-related genes (*BAAT*, *SCTR*, and *CYP7A1*) [35] was significantly higher in the Gel culture system than in the other 3D culture systems. In contrast, the expression of hypoxia-related genes [36,37,38], such as *HK1*, *PFKP*, *TLR4*, and *SLC2A1*, was significantly lower in the HepG2 cells cultured in the Gel system than in the other 3D culture systems, suggesting that necrosis in the other 3D culture systems was associated with hypoxia.

In summary, HepG2 cells in various 3D culture systems maintained the expression of functional hepatocyte markers after prolonged culture. The gene expression pattern of HepG2 cells in the Gel culture system was similar to that in the ULAP and HD systems. Specifically, the Gel culture system exhibited more ECM–receptor interactions, greater levels of bile secretion, a higher drug metabolism rate, and a lower hypoxia-induced necrosis rate than the other 3D culture systems.

## 3. Discussion

The aim of this study was to design a simple and controllable high-throughput-compatible 3D hepatocyte modeling method with better liver properties than current models to improve the overall applicability of HTS studies. We established a hydrogel-based 3D hepatocyte culture system using HepG2 cells specifically designed for high-throughput drug safety screening. We compared this system with other 3D cell culture systems in terms of its hepatotoxicity and metabolic activity.

The preparation of microspheres using high-voltage electrostatic methods is often a simple setup and requires complex handling; the prepared microspheres are usually poorly uniform and difficult to freely control in terms of size [39,40,41]. In this study, we developed a microsphere generation device based on HVE field technology, enabling the rapid and precise preparation of sodium alginate hydrogel microspheres of a uniform and controllable size. By adjusting the distance between the needle tip and the liquid surface (D), injection speed (S), and voltage (V) through the operator interface of the device, we varied the particle size of the hydrogel between 200 and 1500 μm (Figure 2). Considering the potential for core hypoxia and necrosis caused by the large diameter of the hepatocyte spheres within the microspheres [42], we selected specific instrument parameters, including a voltage of 8 kV, a height of 4 cm, and an injection speed of 0.01 mm/s, to ensure rapid and stable microsphere preparation (CV < 5%).

In addition, the hydrogel material in the microspheres is essential for hepatocyte modeling as the extracellular matrix of HepG2 cells. The pores of the material influence cell attachment, growth, and the diffusion of oxygen, nutrients, and metabolites. We obtained different porosities and pore sizes in the material by adjusting the concentration of sodium alginate and the freezing process parameters [43,44]. The pore size directly influences the diffusion of oxygen, nutrients, and metabolites. To prevent core hypoxia in hepatocyte spheroids, we freeze-dried 1.0% sodium alginate at −20 °C for 2 h followed by freezing at −80 °C to prepare hydrogel materials with suitable pore sizes. Additionally, using lower concentrations of sodium alginate made the hydrogel materials softer and promoted increased cytochrome p450 activity and urea production [5]. Our findings indicated that the increased oxidation of sodium alginate resulted in decreased cell viability, while the concentration of RGD grafting had no significant effect on cell viability (Figure 3b). To enhance the biocompatibility, surface activity, and cell adhesion of the materials, we incorporated gelatin and collagen into the hydrogel materials. The results showed that the addition of collagen was more beneficial for the proliferation of HepG2 cells (Figure 3c), likely because collagen better mimics the ECM conditions of hepatocytes in vivo [45,46]. We screened hydrogels for optimal pore size, oxidation level, and engrafted RGD concentration to mimic the in vivo microenvironment and enhance HepG2 cell viability.

To investigate whether the RCHM-based hepatocyte model better reflects in vivo liver physiology, its performance was compared with that of hepatocyte models prepared from ULAPs, HDs, and Scaf. During the early stages of cell culture, scaffold-based models such as RCHM and Scaf models, as well as 2D culture models, exhibited higher proliferation rates than scaffold-free approaches (Figure 4a). Among these cells, the HepG2 cells in the RCHM model showed the fastest proliferation rate, likely due to the mechanical properties of the culture matrix, which affect the impact of cell–matrix interactions on the cell behavior [47,48], as well as the fact that a scaffold-free culture, such as the RCHM model, allows cells to aggregate into clusters, with only the outer layer of cells having sufficient room to proliferate. HepG2 cells cultured on Scaf and in 2D cultures exhibited attenuated increases in their proliferation rates on days 15 and 10, respectively, indicating the unsuitability of these cells for long-term hepatocyte cultures. RCHM, which is similar to the ECM in terms of porosity, mechanical properties, and hepatocyte biocompatibility, allowed the HepG2 cells to maintain a high proliferation rate (121.02 ± 7.49%), even on day 20. Hepatic-drug-metabolizing enzyme activity is an important factor affecting the hepatotoxicity of drugs tested in 3D cell models. The metabolic capacity of the 3D culture models, particularly hepatic drug enzyme activity, was evaluated. The EROD and UGT enzyme activities, representing phase I and phase II metabolic activity, respectively, were measured to examine the hepatic drug enzyme expression in 2D culture and 3D models. Compared with those in the 2D culture system and in the other 3D culture systems, the hepatocyte spheroids cultured in RCHM exhibited greater levels of EROD activity, confirming the favorable hepatocyte biocompatibility of the RCHMs. After 20 days in culture, the EROD and UGT activities of all the 3D hepatocyte culture models, except for those in the Scaf group, were significantly higher than those in the 2D culture group (Figure 4b,c). Furthermore, HepG2 cells in the RCHM, ULAP, and HD groups showed significantly increased levels of albumin synthesis and urea secretion capacity compared to those in the 2D and Scaf groups (Figure 4d,e). The drug sensitivity results revealed that the 3D hepatocyte culture model exhibited a lower level of sensitivity to doxorubicin than the 2D-cultured cells, which was consistent with previous findings [49,50,51]. This outcome is attributed to the dense accumulation of cells in hepatocyte spheroids in 3D cultures, which makes it difficult for the drug to permeate and results in reduced toxicity levels [52,53]. Calcein-AM/PI staining demonstrated that the HepG2 cells in RCHM formed small-diameter cell clusters without necrosis. In contrast, hepatocyte spheroids in the ULAP and HD groups exhibited varying degrees of necrosis due to their large size, which hindered the diffusion of oxygen and nutrients to the spheroids [54]. Additionally, HepG2 cells on Scaf also displayed necrosis, further confirming that this culture method is not suitable for long-term cell cultures. Through the improved proliferation of HepG2 cells by the RCHM system, hepatocyte spheroids can be generated more quickly while hepatocyte markers are broadly expressed. Due to these HepG2 spheroids, the system displays increased levels of physiologic function and metabolic capacity, as well as the ability to maintain and proliferate for at least 20 days, and can be used for repeated dosing to predict long-term hepatotoxicity.

Additionally, we further analyzed the liver-specific functional differences between different 3D culture models at the gene expression level from a bioinformatics perspective. The RNA-seq results demonstrated that the HepG2 cells cultured in the RCHM, ULAP, and HD systems exhibited similar gene expression patterns, with the RCHM and HD cultures showing relatively better levels of sample reproducibility (Figure 6). In comparison to those in the 2D culture system, the HepG2 cells in the 3D culture systems maintained the expression of functional hepatocyte markers even after prolonged cultures. They also exhibited an upregulated expression of genes associated with liver differentiation and metabolism (Figure 7a), including *HNF4α*, *TJP1*, *ARG1*, phase I metabolic enzymes (CYP1A2, CYP3A4, CYP2D6, CYP2E1), and phase II xenobiotic-metabolizing enzymes (UGT1A9). Moreover, the Scaf group showed a reduced expression of certain phase II metabolizing enzyme genes (*UGT2B4*) after 20 days of culture, resulting in a decreased ability to catalyze the 4-MU reaction [55]. Consequently, the Scaf group exhibited lower levels of UGT enzyme activity than those of the other 3D culture systems (Figure 4c). The higher expression of the *ABCB1* gene, which has been linked to tumor multidrug resistance, in the 3D culture system suggests that *ABCB1*-encoded P-glycoprotein (the primary drug efflux transporter) is critical for the reduced sensitivity to doxorubicin in the 3D culture systems compared to the 2D culture system [56]. In addition, increased doxorubicin chemoresistance was also associated with higher levels of CYP3A4 enzyme expression in a 3D culture system [57]. A transcriptome analysis indicated that genes related to ECM–receptor interaction were more highly expressed in the RCHM culture system (Figure 7c), suggesting that the increased drug resistance is also accompanied by ECM remodeling, consistent with previous studies [33,34]. Additionally, the RCHM culture system exhibited higher levels of bile secretion and drug metabolism activity than those of the other 3D culture systems. Furthermore, the low expression of hypoxia-associated genes further indicated that a sufficient oxygen supply was generated in the RCHM culture system, which supported prolonged cultures of hepatocyte spheroids, as it prevented necrosis in the central region of the spheroids due to hypoxia.

## 4. Materials and Methods

### 4.1. Microsphere Generation Device

The proposed microsphere generation device is depicted in Figure 1a. Within the working chamber (Figure 1b), a Petri dish filled with calcium chloride (Aladdin, Shanghai, China) solution sits atop a negatively charged plate. A 25G injection needle (Musashi, Japan) mounted on the mechanical arm was connected to the positive pole and driven by No. 1 motor (SAMSR, Tokyo, Japan) to control the distance between the needle tip and the salt solution level (D). The injection speed (S) of the syringe (Mishawa, Shanghai, China) was also precisely controlled by the propeller drive of the No. 2 stepper motor, and syringe was connected to the injection needle through a catheter. A high-voltage power supply (Matsusada, Otsu, Japan, 0~20 kV) generated a stable HVE field between the needle and the negative electrode plate (Figure 1c). Briefly, a mixture of hydrogel and HepG2 cells was dropped using a syringe into a Petri dish containing a solidified solution, and the droplet size was influenced by the electric field force. The device is operated through a touch screen interface, enabling control of the output voltage (V), working distance (D), and injection speed (S) to vary the electric field force.

### 4.2. Parameters for Microsphere Preparation

A 1 mL syringe was drawn of 1% sodium alginate solution and placed in front of the No. 2 motor. Additionally, 6 mL of a calcium chloride solution containing 0.1% Tween 20 was added to the Petri dish, which was then positioned in the device as shown in Figure 1. The experiment focused on investigating the influence of the voltage (V), the distance between the needle tip and the liquid surface (D), and the injection speed (S) on the particle size of the microspheres. The device was configured with the following parameters: (1) D = 4 cm, S = 0.01 mm/s, and V varied from 4, 5, 6, 7, 8, 9, 10, 12, 14, 16, 18, and 20 kV; (2) S = 0.01 mm/s, V = 8 kV, and D was adjusted to be 1.5, 1.6, 1.7, 2, 3, 4, 5, 6, 7, and 8 cm; and (3) D = 4 cm, V = 8 kV, and S was set to be 0.001, 0.005, 0.01, 0.02, 0.04, 0.06, 0.08, 0.1, 0.2, 0.4, 0.8, 1, 2, and 4 mm/s. The microspheres produced were photographed under a microscope (Nikon Ti2-u) at ×100 magnification, and the diameter of microspheres was measured using ImagePro Plus version 6.0.

### 4.3. The Freeze-Drying Process used for Generating Material with Different Pore Sizes

To optimize the freeze-drying process, 0.5%, 1%, 1.5%, and 2% alginate solutions were prepared and subjected to freezing at three different temperatures. The samples were subjected to cooling at ① −20 °C, ② −80 °C, or ③ −20 °C for 2 h followed by freezing at −80 °C. Subsequently, the materials were lyophilized for 50 h at −52 °C and 0.030 mbar using a lyophilizer. The resulting lyophilized materials were examined and compared via scanning electron microscopy (SEM, GeminiSEM 300, ZEISS, Oberkochen, Germany) to assess their structural characteristics. SEM images were analyzed using ImagePro Plus to measure pore size by calibrating the line measurement tool to the image scale [58].

### 4.4. Preparation of Bioactive Hydrogels

To prepare hydrogel samples, 1000 µL, 500 µL, or 250 µL of 0.25 M sodium periodate was added to 50 mL of a 1% sodium alginate solution. These samples were sodium alginate with high, medium, and low levels of oxidization and are labeled A, B, and C, respectively. The mixture was gently shaken on ice for 1 h. The reaction was stopped by the addition of ethylene glycol, followed by the addition of 0.15 g of NaCl. The resulting solution was then dialyzed using an equal volume of anhydrous ethanol and subjected to lyophilization. Three oxidized sodium alginate solutions (1%) were prepared by incorporating 25 μg/mL and 50 μg/mL RGD peptide into each sample. After 1 h of RGD treatment, the partially oxidized sodium alginate samples were reduced by the addition of an appropriate amount of sodium borohydride and incubated in an ice water bath for 2 h, which immobilized the RGD peptide. The samples were then placed in a refrigerator set to 4 °C, where they were subjected to dialysis. After dialysis, the samples were freeze-dried.

### 4.5. Analyzing the Bioactivity of Different Sodium Alginate Solutions

HepG2 cells (CSTR:19375.09.3101HUMSCSP510, the Cell Bank, Chinese Academy of Sciences, Shanghai, China) were cultured in DMEM (Gibco, Waltham, MA, USA) supplemented with 10% fetal bovine serum (FBS; Gibco, Waltham, MA, USA) and a 1% penicillin/streptomycin solution (Biyuntian, Shanghai, China) at 37 °C in a cell incubator with 5% CO_2_.

For each of the six modified sodium alginate solutions, a concentration of 2% was prepared. A HepG2 cell suspension was mixed with a sodium alginate solution at a 1:1 ratio, which resulted in a final concentration of 15 × 10^5^/mL of HepG2 cells in solution. The mixture was then used to generate homogeneous cell-encapsulated microspheres with a 400 μm diameter using the microsphere generation device under aseptic conditions. In a 96-well plate, microspheres were added to each well (equivalent to 5000 cells/well), and four replicate wells were established for each sample. The microspheres were cultured in 200 μL of DMEM containing 10% FBS and incubated at 37 °C in a cell incubator with 5% CO_2_. On days 5 and 10, the hydrogels were degraded using a sodium citrate solution to release the cell clusters. To assess cell viability, 100 μL of CellTiter-Glo^®^ 3D solution (Promega, Beijing, China) was added to each well and thoroughly mixed for 5 min to lyse the cells. The plate was then incubated at room temperature for 25 min to stabilize the luminescence signal, and the luminescence value was determined with a multifunctional plate reader (Thermo Fisher, Shanghai, China, Varioskan LUX).

### 4.6. Hydrogel Formulation Selection Based on Cell Bioactivity

A 2% solution of ROSAH was mixed with a HepG2 cell suspension and collagen (0.2%) or gelatin (10%) at a ratio of 2:1:1 to form cell-encapsulated microspheres. The proliferation of the cells was assessed on days 1, 3, 6, 10, 15, and 20 using a CCK-8 assay kit (Biyuntian, Shanghai, China). The absorbance (optical density, OD) of each well was measured at 450 nm using a multifunctional plate reader, and the corresponding values were recorded and calculated.

### 4.7. Establishment of 3D Hepatocyte Models

In this study, to compare different 3D culture methods for establishing hepatocyte models using HepG2 cells, we prepared a 3D hydrogel culture system by mixing ROSAH (2%), collagen (0.2%), and a cell suspension at a ratio of 2:1:1. Microspheres were formed with this mixture and placed in 96-well plates. In addition, we used two simple and common scaffold-free methods: ULAPs (Spheroid Microplate, CORNING, Shanghai, China) [59], HDs (InSphero Gravity, Schlieren, Zurich, Swiss) [60], and another scaffold-based method: PS fibrous scaffolds (3D Biotek, Warren, NJ, USA) [61]. HepG2 cells were cultured according to the respective manufacturers’ instructions, and a 2D HepG2 cell culture in 96-well plates was used as the control. All five cultures were seeded at a cell density of 5000 cells per well and incubated at 37 °C in a cell incubator with 5% CO_2_.

### 4.8. Cell Proliferation Assay

Cell proliferation was assessed 1, 3, 6, 10, 15, and 20 days after model establishment using a CCK-8 assay kit [62], for which hydrogel group was measured after releasing the cell mass. The culture medium was replaced with a serum-free culture medium containing CCK-8. After 2 h of culturing, 100 μL of culture was taken from each well and then transferred to a new 96-well plate. The optical density (OD) value of each well was measured at 450 nm using a multifunctional plate reader and the OD values of the first day of each culture system were used as the initial value (0%) to calculate the cell proliferation rate.

### 4.9. CYP Activity Assays

An EROD assay is used primarily to evaluate the enzymatic activity of human CYP1A1 [63]. To activate EROD, 100 μM omeprazole was added to HepG2 cells in culture and incubated for 48 h. Subsequently, 2 μM 7-ethoxyresorufin in Hanks’ balanced salt solution (HBSS) (pH 7.4) was added to the cultured cells and incubated for 1 h at 37 °C. Then, the mixture was removed from the cell culture plate and transferred to a black 96-well plate, and the fluorescence intensity was measured at an excitation wavelength of 535 nm and an emission wavelength of 585 nm using a multifunctional plate reader. The concentrations of the samples were determined on the basis of standard curves established with resorufin (Sigma, Shanghai, China) at concentrations ranging from 0 to 1000 nM. The enzymatic activity was normalized based on the protein content in the cells, and the results are expressed as pmol/mg·h.

### 4.10. UGTs’ Activity Assay

For the measurement of UGT activity, the substrate 4-MU was quantified after its incubation with cells [17]. Briefly, 100 μM 4-MU in 100 μL of HBSS was incubated with the cells for 1 h. The supernatant was then transferred to a black 96-well plate, and standard curves were generated using 4-MU concentrations ranging from 0 to 100 μM. To achieve a pH of 11, 4 μL of 0.1 M NaOH was added to each well, and the fluorescence was measured at an excitation wavelength of 340 nm and an emission wavelength of 460 nm. The data are expressed as nmol/10^4^ cells·h.

### 4.11. Albumin and Urea Secretion Assays

After 5, 10, 15, and 20 days in the culture systems, culture supernatants were collected, the albumin content was measured using a human albumin ELISA kit (Solarbio, Beijing, China), and the urea content was measured using a urea nitrogen test kit (Nanjing Jiancheng, Nanjing, China). The albumin data are expressed as ng/10^4^ cells·h, and the urea data are expressed as μg/10^4^ cells·h.

### 4.12. Drug Sensitivity Analysis

After 10 days of culture, the cells were treated with varying concentrations (0, 0.001, 0.01, 0.1, 1, 10, 100, 500, and 1000 μg/mL) of doxorubicin in DMEM complete medium. The 2D cells were used as the control group. After 48 h, cell viability was assessed using a CCK-8 assay kit. Cell-free wells were used as blank controls. The luminescence values were recorded with a multifunctional plate reader, and the cell viability was calculated by comparing the luminescence values of each culture method group to that of the respective untreated group control, which was set at 100%.

### 4.13. Cell Viability Assay

After 20 days of cell culture, microcellular spheroids cultured in hydrogels and ULAPs, HDs, and Scaf with attached growing cells were transferred to confocal dishes. The microcellular spheroids were washed three times with PBS and then incubated with 4 μM Calcein-AM (Biyuntian, Shanghai, China) and 5 μM PI (Biyuntian, Shanghai, China) at 37 °C for 45 min in the dark. Subsequently, they were washed three times with PBS and stained with DAPI staining solution for 5 min in the dark. The images of multicellular spheroids were obtained using laser confocal microscopy at excitation wavelengths of 490 nm and 535 nm and emission wavelengths of 515 nm and 617 nm for Calcein-AM and PI, respectively.

### 4.14. RNA-Seq Analysis

Total RNA was extracted from HepG2 cells cultured in 2D for 5 days and in each of the four different 3D culture systems for 20 days using TRIzol (Thermo Fisher, Shanghai, China). Libraries were constructed and three biological replicates per group were sequenced using a NovaSeq 6000 system (Illumina Inc., San Diego, CA, USA). The read data were processed and analyzed using HISAT2 (v2.0.5), featureCounts (v1.5.0-p3), and DESeq2 (v1.20.0) with the hg38 reference genome and gene annotation Ensembl GRCh38. To account for sequencing depth and gene length, we report gene expression values as fragments per kilobase million (FPKM). We calculated the square of Pearson correlation coefficients (R2) with the FPKM values of all genes for each sample. FPKM greater than 1 indicates gene expression, and a Wayne diagram was used to show the overlap of gene expression between the different groups. The criteria for differential gene screening were set as |log2(FoldChange)| ≥ 1 and padj ≤ 0.05. We utilized clusterProfiler (v3.8.1) software to determine the DEGs that were significantly enriched in a KEGG pathway.

### 4.15. Statistical Analysis

The results of the statistical analyses are presented as the mean ± standard error of the mean (SEM). Differences between groups (multiple means data) were estimated by one-way analysis of variance (ANOVA), followed by a post hoc multiple comparison (Tukey) test. Statistical analyses were performed using GraphPad Prism 5.0. Statistical significance was indicated with an asterisk (*) when the *p* value was less than 0.05.

## 5. Conclusions

In summary, we developed a new hydrogel system (RCHMs) suitable for long-term cell cultures using HepG2. A device based on an HVE field was used to produce controllable and uniformly sized microspheres for 3D cultures of HepG2 cells, and peculiar characteristics of hepatocytes were seen. Compared to 2D and other 3D culture methods, this 3D culture system with an ECM microenvironment has better levels of cell viability, metabolic capacity, ECM remodeling, and bile secretion. Therefore, this culturing approach may also be suitable for hepatocytes. This 3D culture system is both controllable and reproducible, exhibiting the potential for the high-throughput screening of long-term drug-induced hepatotoxicity. However, further validation with a substantial number of compounds is still needed. Furthermore, the development of new models using hepatocytes co-cultured with other hepatocytes such as Kupffer cells and stellate cells could further improve liver characteristics and drug toxicity predictions in humans.

## Data Availability

Data is contained within the article.
